# Bacterial Community Composition in the Growth Process of *Pleurotus eryngii* and Growth-Promoting Abilities of Isolated Bacteria

**DOI:** 10.3389/fmicb.2022.787628

**Published:** 2022-01-31

**Authors:** Liding Chen, Miao Yan, Xin Qian, Ziwei Yang, Yanfei Xu, Tianjiao Wang, Jixuan Cao, Shujing Sun

**Affiliations:** College of Life Sciences, Fujian Agriculture and Forestry University, Fuzhou, China

**Keywords:** bacterial diversity, community composition, endophytic bacteria, physiological function, *Pleurotus eryngii*

## Abstract

The effects of biological factors on the vegetative growth process of mushrooms remain largely unexplored. We investigated the bacterial community in different growth stages of *Pleurotus eryngii* by high-throughput sequencing technology to explore the relationship between interacting bacteria and the growth and development of *P. eryngii.* We found significant variances in mushroom interacting association bacteria (MIAB) compositions among the samples from different growth stages, and 410 genera were identified. The bacteria in the full-bag and post-ripe stages were shifted to the biocontrol and growth-promotion ones. The mushroom growth-promoting bacteria (MGPB) were also isolated successfully and identified as *B. cereus* Bac1. The growth speed and density of mycelial pellets of *P. eryngii*, and activities of two exoenzymes (laccase and amylase), were analyzed by adding the different volumes of cell-free fermentation broth of *B. cereus* Bac1 to fungal culture media. The results showed that when a 5 mL cell-free fermentation broth was used, the growth speed of *P. eryngii* hyphae was enhanced by 1.15-fold over the control and reached 0.46 mm/h. The relative activity of laccase and amylase was increased by 26.9 and 43.83%. Our study revealed that the abundant interacting bacteria coexist with *P. eryngii* hyphae. Moreover, the abundance of some bacteria exhibiting a positive correlation with the growth periods of their host fungi can effectively promote the growth of the host, which will provide technical supports on the high-efficiency production of *P. eryngii* in factory cultivation.

## Introduction

In general, the growth and development of edible fungi are divided into the vegetative growth phase and reproductive growth phase. Some cultivation environmental factors, such as light, temperature, moisture, and CO_2_, can influence the growth and development of edible fungi at these two phases through different metabolic pathways (Tisch and Schmoll, 2010; Lenoir et al., 2016). In addition to the cultivation environment, biological factors can also be a pivotal part of the production cycle of edible fungi. As hosts, edible fungi interact with many beneficial microorganisms (Carrasco and Preston, 2020). These interacting microorganisms play crucial roles in improving nutrient uptake, promoting, or inhibiting the growth of host fungi, and preventing pathogen contamination (Frey-Klett et al., 2007; Noble et al., 2009; Aslani et al., 2018; Dygico et al., 2019).

Research on mushroom-interacting microorganisms has now identified many bacteria that may play a role in promoting hyphal extension and increasing compost productivity (Kertesz and Thai, 2018). In our former research (Sun et al., 2020a), many mushroom interacting association bacteria (MIAB) in the cultivation bags in different growth stages of mushrooms were detected and classified using high-throughput sequencing technology. These bacteria can effectively promote the growth and metabolism of mushrooms. The diversity and community structure of bacteria can reflect the growth and nutrient utilization status of mushrooms. At the same time, the mushroom growth-promoting bacteria (MGPB) were isolated, and the growth promotion effects were systematically analyzed. *P. putida* was the best growth-promoting inoculant among 23 tested bacterial strains that could increase the fruiting-body yield of *Agaricus bisporus* (Zarenejad et al., 2012). The bioinoculant of *Glutamicibacter arilaitensis* MRC119 can be potentially used as an eco-friendly substitute improving the fruitbody yields and biological efficiency of oyster mushrooms (Kumari and Naraian, 2021). *Pseudomonas fluorescens* strains could promote the formation of the primordium, the mycelial growth, and the fruiting body productivity of *Pleurotus eryngii* (Kim et al., 2008) and *Pleurotus ostreatus* (Cho et al., 2003).

The fermentation broth of beneficial bacteria also contributed to promote the development of edible fungi. For example, the harvest time of *P. eryngii* was ahead of schedule due to the increase of the mycelia growth rate raised by adding the fermentation broth of *Pseudomonas* sp. P7014 to the growing substrate (Kim et al., 2008). Although the increasing knowledge of microbiome in soil or casing layer of edible fungi habitats, such as *Morchella sextelata* (Benucci et al., 2019), *Stropharia rugosoannulata* (Gong et al., 2018), *Tuber melanosporum* (Deveau, 2016) and *Agaricus bisporus* (Siyoum et al., 2016), microecology in cultivation bags of mushroom factory production remains less studied. Conclusions drawn from these literatures indicate that the interaction between edible fungi and beneficial microorganisms is universal and beneficial to the production of edible mushrooms.

It is crucial to delve into the composition and function of beneficial bacteria in the growth media of non-casing soil edible fungi, to explore potential interactions between the edible fungi and bacteria, and to further improve mushroom production efficiency and quality. *P. eryngii* (DC.ex.Fr.) Quel. belongs to the family *Pleurotaceae* and genus *Pleurotus*. It is ranked second among the commercially cultivated mushrooms in China (Stajic et al., 2009) due to its undemanding cultivation, good taste, and rich nutrition. In this study, to improve the productivity and quality of *P. eryngii* in factory model cultivation, the microbial diversity of samples obtained from the cultivation bags of *P. eryngii* was determined by high-throughput sequencing technology. The functions of the isolated bacteria were preliminarily examined. Some substrate-associated bacteria were discovered, and their essential role was also confirmed in promoting the growth of *P. eryngii* hyphae. These interacting bacteria may be developed into useful agronomical amendments to increase mushroom productivity through growth promotion. The present work will display the first evidence to prove the existence of the abundant interacting bacteria during the growth process of *P. eryngii*, which will lay the theoretical basis and provide technical supports for the high-efficiency production of *P. eryngii* with good quality.

## Materials and Methods

### Media

The strain *P. eryngii* is a strain cultivated by Lvshengyuan Biotechnology Co., Ltd (Zhangzhou, Fujian Province, China). The strain was maintained on potato dextrose agar medium (potato 200 g, glucose 20 g, peptone 3 g, yeast powder 3 g, KH_2_PO_4_ 1.5 g, MgSO_4_⋅7H_2_O 1.5 g, agar 20 g, H_2_O 1 L) at 25°C. The bacteria were cultivated in LB medium (yeast powder 5 g, beef extract 5 g, peptone 10 g, NaCl 10 g, agar 20 g, pH 7.2∼7.4). All these media were prepared by homemade raw materials.

### Sample Collections

Cultivation bags of *P. eryngii* were collected from Lvshengyuan Biotechnology Co., Ltd (Zhangzhou, Fujian Province, China). The growing substrate formulation (all ingredients based on dry substrate weight, w/w) consisted of 25% sawdust, 27% corncob powder, 13% bagasse, 15% bran, 10% bean pulp, 8% corn starch, 1% light calcium carbonate, and 1% calclime. All mixed substrates were sterilized with high-pressure steam under constant monitoring for 2.5 h at 135^°^C. Cultivating substrates at different hyphal growth stages of *P. eryngii* were sampled from the cultivation bags. Three different growth stages are PEBH (the half-bag of *P. eryngii* hypha stage), PEBF (the full-bag of *P. eryngii* hypha stage), and PEBM (the post-ripe stage) because these three different growth stages are extremely representative in the life history of *P. eryngii*. At these three different growth stages, a lot of MIAB in the cultivation bags can be detected and classified systematically and the diverse functions of different bacterial communities can be investigated and clarified in detail. Most of all, the mushroom growth promoting bacteria (MGPB) can be isolated and the growth promotion effects can be systematically analyzed at these three different growth stages. During the experiment, samples (5 g each) were collected from the top, middle, and bottom of each bag using aseptic techniques to make the samples representative of the microorganism population. Sampling was repeated in three independent bags. Three samples from the same part of three independent bags were immediately mixed and frozen by liquid nitrogen and then used for DNA extraction and microbial diversity analysis.

### DNA Extraction and Illumina HiSeq 2500 Sequencing

The 16S rDNA library preparations and Illumina HiSeq 2500 sequencing were performed at Biomaker, Inc. (Beijing, China). According to the manufacturer’s instruction, DNA was extracted from 0.4 g of samples from cultivation bags using EasyPure Genomic DNA Kit (TransGen, Beijing, China). The bacterial 16S rDNA gene was amplified with the forward primer 338 F (5′-ACTCCTACGGGAGGCAGCA-3′) and the reverse primer 806 R (5′-GGACTACHVGGGTWTCTAAT-3) targeting the V3 and V4 regions (Sun et al., 2020a). When amplifying the bacterial 16S rDNA gene, PCR thermal cycle profile was as follows: 2 min at 98°C, 30 cycles of 30 s at 98°C, 30 s at 50°C, and 1 min at 72°C, a final extension at 72°C for 5 min, and hold 4°C.

### High-Throughput Sequencing Data Analysis

Amplicons were sequenced using a paired-end method by Illumina HiSeq. Raw data generated from the high-throughput sequencing run were joined using the FLASH v1.2.11, filtered using the Trimmomatic software (V 0.33), and removed the chimera sequences using the UCHIME software (V8.1). Only the effective tags classified into OTUs will be pre-clustered to 97% sequence identity using the software QIIME v1.8.0 (Morey et al., 2013).

The OTUs were assigned into taxonomic categories using the ribosomal database program (RDP) with a confidence threshold of 0.8 and predicted to the kingdom, phylum, class, order, family, genus, and species level using the Silva database. The alpha diversity index including ACE value, Chao1, Simpson index, and Good’s coverage were calculated using “vegan” R package. One-way analysis of variance (ANOVA) and subsequent *post hoc* Tukey’s honestly significant difference (HSD) tests were used to compare differences in OTU richness among different periods. Chord diagram and heatmap were plotted to investigate abundant taxa at phylum and genus levels using “circlize” and “pheatmap” R packages, respectively. Then, principal coordinate analysis (PCoA) based on Bray–Curtis distance was performed to investigate the patterns of the bacterial community structure. In addition, analysis of composition of microbiomes (ANCOM) test (Mandal et al., 2015; Kaul et al., 2017) were used to explore the significant differences in the relative abundance at the genus level among the different groups. We generated a table grouping the sequences taxonomically assigned to the same genus according to the report (Ramirez et al., 2021) before the implementation of ANCOM.

### Strains Isolation From *Pleurotus eryngii* Hypha

*Pleurotus eryngii* hyphae were sampled from cultivation bags, washed by sterilized water, and ground in a sterile environment. Serial dilutions of the hyphae were prepared with sterilized pure water. A total of 0.1 mL of the dilutions was spread on LB agar media plates. The samples were incubated at 35°C for 48 h to enumerate and isolate the microbic colonies. The bacterial 16S rRNA gene was amplified with the forward primer 16S-F (AGAGTTTGATCCTGGCTCAG) and the reverse primer 16S-R (GGTTACCTTGTTACGACTT) (Sun et al., 2020a).

### Cell-Free Fermentation Broth Preparation

The culture of isolated bacteria was grown in LB liquid medium and incubated on an incubator shaker at 37°C and 180 r⋅min^–1^ until the OD_600_ reached 0.6∼0.7 after dilution two-fold. The culture then was centrifuged (at 10 000 r⋅min^–1^ and 4°C for 20 min) and filtered (Φ = 0.45 μm) to obtain a cell-free fermentation broth.

### Cell-Free Fermentation Broth Assay

The modified media were prepared by mixing different volumes of cell-free fermentation broth into sterilized PDA enrichment media. Then the media poured into a petri dish. The additive amounts of culture and fermentation broth were 1, 3, 5, 7, 9, and 11% (v/v). Discs of *P. eryngii* hyphae (Φ = 6.0 mm) were inoculated in the middle of modified media and grew at 25°C. The diameters of *P. eryngii* hyphae and their growth rates were measured when they grew over the entire plate (Zhao et al., 2007). The average of three diameter measurements was used to estimate mycelial growth rate, and the mycelial growth rate was expressed as a mean diameter of the increase in mycelia length. The measurement and calculation were repeated in triplicate. When submerged fermentation was carried out, the same additives of cell-free fermentation broth were added to sterilized liquid PDA enrichment media. Seven mycelial agar discs were cut from *P. eryngii* hyphae (Φ = 5.0 mm) into each flask. The strain was cultivated in a rotary shaker at 120 r⋅min^–1^, at 25°C, and was sampled each day for 7 days. The density of *P. eryngii* hyphae pellets and activity of laccase and amylase in the fermentation broth was measured as follows: 4.0 mL of *P. eryngii* mycelial broth was transferred to a glass Petri dish, where the number of mycelial pellets was counted. The experiment was repeated three times. The average of the three replicates was used to represent the density of *P. eryngii* hyphae pellets. According to the method established by Sun et al. (2020b), laccase activity was determined by monitoring the rate of 5 mM 2,2′-azino-bis(3-ethylbenzthiazoline-6-sulfonic acid) (ABTS) oxidizing to its cation radical (ABTS^+^) at 436 nm (ε_*4*36_ = 29,300 M^–1^ cm^–1^) in 0.1 M sodium acetate buffer (pH 5.0) at 30°C. Briefly, the 3 mL reaction system consisted of 2.4 mL of HAc-NaAc buffer solution, 0.2 mL of ABTS solution, and 0.4 mL of crude enzyme. The mixture was preheated in a 30°C water bath for 5 min, and the changes in absorbance at 436 nm for the first 3 min were recorded after zeroing the spectrophotometer. One unit (U) of enzyme activity was defined as the amount of enzyme required to oxidize 1 μmol of ABTS per minute at 30°C.

The amylase activity assay was conducted using the 3,5-dinitrosalicylic acid (DNS) method (Miller, 1959). Briefly, 1.0 mL of sample and an equal amount of substrate (1.0% w/v soluble starch) were mixed thoroughly, and test tubes were incubated at 37°C for 15 min in a water bath. After 10 min, the reaction was stopped by adding 2.0 mL of DNS reagent, and tubes were kept in a boiling water bath for 5 min. Tubes were cooled at room temperature, and absorbance was measured at 540 nm against substrate and enzyme blank. One unit (U) of amylase activity was defined as the amount of enzyme that releases 1 μmol of reducing sugar as D-glucose per min under the assay conditions. Extracellular protein concentration was estimated using the Lowry method and bovine serum albumin as the standard.

### Statistical Analysis

All experiments were performed in triplicate to ensure the precision of the results. Data analysis was performed with SPSS software. The difference between samples was estimated using a one-way analysis of variance (ANOVA) to test for the significant differences between mean ± SD of treatments (*n* = 3) at *p* < 0.01 significance level.

## Results and Discussion

### Sequencing Data Analysis

**FIGURE 1:**
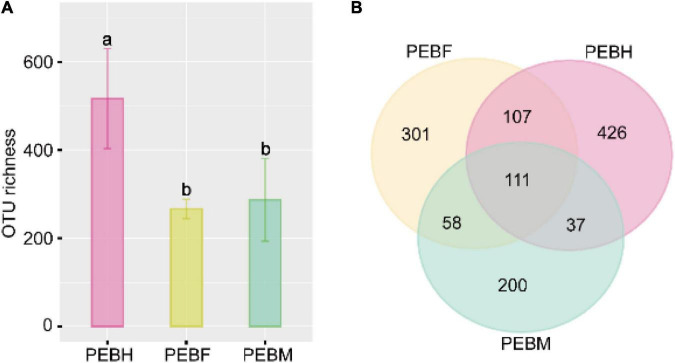
The OTU number of samples from cultivation bags **(A)** and Venn analysis at different periods **(B)**. Different letters on bars indicate statistical significance in OTU richness between groups by Tukey’s HSD test at *P* < 0.05.

In this study, Illumina HiSeq 2500 was used for its incredible speed and throughput, unprecedented flexibility, exceptional data quality, and complete end-to-end sequencing solutions. A total of 457,104 pairs of reads were generated from the high-throughput sequencing runs. After quality control, an average of 42,374 effective tags was recovered from nine samples (three samples per stage). We removed OTUs that do not belong to bacterial taxa and have fewer than 0.005% relative abundance. Then, we rarefied each sample to the minimum size (17,948), resulting in a normalized dataset comprising 1,240 bacterial OTUs. As shown in Figure 1A, the average OTUs numbers varied at different growth periods of *P. eryngii* hyphae. The maximum OTUs were generated at the half-bag stage of the *P. eryngii* hyphae (PEBH), which was significantly more than OTUs of PEBF (the full-bag stage) and PEBM (the post-ripe stage). PEBM has more OTUs numbers than PEBF, but not statistical significance. It is interesting to note that 111 OTUs were shared among three growth stages of *P. eryngii* (Figure 1B). When the growth shifted from the half-bag to the full-bag stage, 218 OTUs were preserved and shared among groups, and 359 new OTUs were generated. When the growth shifted from the full-bag stage to the post-ripe stage, the numbers of shared OTUs and newly generated OTUs were changed to 169 and 237, respectively. These results indicated that the cultivation bags have different bacterial communities coexisting with *P. eryngii* hyphae at various growth periods.

The overall perception of “microecologics” has been around. Currently, edible fungi can no longer be considered an independent population because most evidence pointed out the symbiosis between bacteria and mushrooms (Li et al., 2015; Siyoum et al., 2016; Gong et al., 2018; Benucci et al., 2019). It is well known that the edible mushrooms displaying above-ground fruit bodies and under-ground hyphae assets can be colonized with diverse bacteria due to the biodiversity of air and soil (Rousk et al., 2010; Zagriadskaia et al., 2013; Deveau, 2016). However, little research has been done on interacting bacteria in cultivation bags full of culture substrates of *P. eryngii.* Therefore, the high-throughput sequencing technologies were used to better understand the whole micro-ecosystem of the cultivation bags during growth periods of *P. eryngii* hyphae. Although only 3 samples per stage were used in the high-throughput sequencing step, we still got pretty good results because all four reversible terminator-bound dNTPs are present in each sequencing cycle and this feature minimizes bias of incorporation and greatly reduces raw error rates in HiSeq 2500 System.

### Analysis of Alpha Diversity

The average coverage of bacteria in the samples from cultivation bags was between 0.9974 and 0.9997 (Table 1), indicating that the sequencing depth was sufficient and the sampling was reasonable. The results could reflect the bacterial community composition in the cultivation bags. The microbial species richness was estimated and compared by Chao1, ACE, and Simpson indices. The order of the ACE and Chao indices at the half-bag period (PEBH) was significantly higher than those at the full-bag period (PEBF) and post-ripe period (PEBM), and PEBH showed the lowest Simpson value among the three periods (Table 1). These results indicated that the bacterial diversity at the half-bag period of *P. eryngii* hyphae was higher than those at the other periods, suggesting that the bacterial diversity in the samples was closely related with the growth process of *P. eryngii* hyphae.

**TABLE 1:** Diversity analysis of bacterial communities at different growth periods.

Group	ACE	Chao1	Simpson	Coverage
PEBH	533.936 ± 123.642a	542.137 ± 121.965a	0.945 ± 0.017b	0.9974 ± 0.0014a
PEBF	270.009 ± 20.198b	269.658 ± 19.649b	0.986 ± 0.002a	0.9997 ± 0.0001a
PEBM	316.834 ± 67.715b	314.543 ± 80.501b	0.969 ± 0.013ab	0.9990 ± 0.0008a

*Different letters indicate statistical significance in alpha diversity between groups by Tukey’s HSD test at P < 0.05.*

### Comparison of Bacterial Communities in the Samples

**FIGURE 2:**
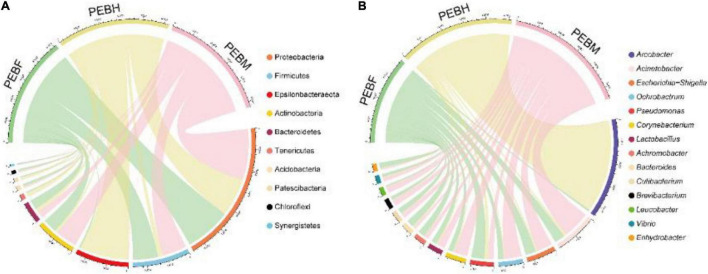
Chord diagram visualizing relative abundances of the dominant bacterial phyla **(A)** and genera **(B)** in different growth stages of *P. eryngii*. The ten most abundant phyla and the genera with relative abundances of > 1% were showed.

Our previous study demonstrated a correlation between the community structure of interacting bacteria and the growth and development of edible fungi (Sun et al., 2020a). On this basis, a novel strategy was developed to improve the industrial production of edible fungi using interacting microbial resources. Therefore, the detailed analysis of the microbiome of *P. eryngii* in production bags will provide an important theoretical basis for a deeper understanding of the existence of the abundant interacting bacteria and the knowledge of interactions of the fungus with other microorganisms. And on top of that, the fungus production technology can be improved by regulating microbial communities after confirming their growth-promoting effects. In the present study, the bacterial community in the samples from cultivation bags was examined by RDP. The 1,240 OTUs generated from the samples were classified into 37 phyla, 74 classes, 151 orders, 228 families, and 410 genera. As shown in Figure 2A, the top 10 abundant phyla were Proteobacteria (34.65∼46.28%), Firmicutes (4.58∼22.91%), Epsilonbacteraeota (0.46∼44.30%), Actinobacteria (2.52∼19.66%), Bacteroidetes (3.50∼8.81%), Tenericutes (0.10∼3.91%), Acidobacteria (0.48∼2.28%), Patescibacteria (0.23∼2.15%), Chloroflexi (0.44∼1.63%) and Synergistetes (0.09∼1.15%), accounting for 96.51% of the total bacteria in three samples. Except for the two phyla Firmicutes and Actinobacteria, other dominant bacteria phyla included Gram-negative bacteria. Throughout the three periods of hyphal growth, phylum Proteobacteria was the second and the first dominant phyla at PEBH and the latter two growth periods of *P. eryngii* hyphae, respectively, mainly including classes Alphaproteobacteria, Gammaproteobacteria and Deltaproteobacteria. The class Alphaproteobacteria was significantly (*P* < 0.01) increased from 2.61 to 12.86% when the growth of *P. eryngii* hyphae switched from PEBH period to PEBF period. However, Campylobacteria, the dominant class in the phylum Epsilonbacteraeota was significantly (*P* < 0.001) decreased from 44.39% to 1.39%. Besides, Firmicutes, Actinobacteria, and Acidobacteria levels were significantly increased up to 5.00, 5.13, and 4.76-folds from PEBH to PEBF. The fourth-highest abundant phyla Actinobacteria was significantly increased from 12.96% in the PEBF sample to 19.67% in the PEBM sample.

A total of 410 genera were identified across the samples, which revealed that bacteria in the cultivation bags were plentiful and varied. The dominant genera (relative abundances > 1%) in the samples was shown in Figure 2B. Those 14 abundant genera were shared by the samples at the three growth periods. Arcobacter was the most abundant genus, whose relative abundance was harply decreased to less than 0.15% at PEBM, from 40.31% at PEBH. Four genera (*Pseudomonas, Escherichia shigella*, *Acinetobacter* and *Bacteroide*) were dominant in PEBF samples with a relative abundance higher than 3.0%. Whereas, the other five genera (*Acinetobacter*, *Ochrobactrum*, *Escherichia-Shigella*, *Corynebacterium*, and *Lactobacillus*) were dominant in PEBM samples. The relative abundance of some genera such as *Acinetobacter, Ochrobactrum*, and *Corynebacterium* was significantly increased up to 2.6∼3.1 folds from PEBF to PEBM. Additionally, ANCOM results showed that some abundant genera including *Acinetobacter*, *Escherichia-Shigella*, *Corynebacterium*, *Achromobacter*, *Cutibacterium*, and *Brevibacterium* were also the important differential generic indicators structuring the bacterial composition among the different periods (Table 2).

**TABLE 2:** ANCOM test results showing the 20 most significant the differences in abundance levels of bacterial genus among the three different periods.

Percentile	0	25	50	75	100	0	25	50	75	100	0	25	50	75	100		

Genus	PEBH	PEBH	PEBH	PEBH	PEBH	PEBF	PEBF	PEBF	PEBF	PEBF	PEBM	PEBM	PEBM	PEBM	PEBM	W	Reject null hypothesis
*Thiovirga*	405	475.5	546	620	694	1	1	1	1	1	1	1	1	2	3	90	TRUE*
*Chrysiogenes*	122	139	156	170	184	1	1	1	1	1	1	1	1	1	1	81	TRUE*
*Porphyromonas*	1	1	1	1	1	1	1	1	1	1	41	54	67	68.5	70	81	TRUE*
*Parabacteroides*	2	4	6	8.5	11	33	34	35	45.5	56	1	1	1	1	1	79	TRUE*
*Oceanobacter*	114	116.5	119	119.5	120	1	1	1	1	1	1	1	1	1	1	73	TRUE*
*Denitrovibrio*	105	108.5	112	119.5	127	1	1	1	1	1	1	1	1	1	1	71	TRUE*
*Cutibacterium*	18	25.5	33	41.5	50	328	380.5	433	460	487	415	416.5	418	423	428	70	TRUE*
*Streptomyces*	1	1	1	1	1	38	41	44	48	52	1	1.5	2	2	2	68	TRUE*
*Sulfurovum*	82	92	102	122	142	1	1	1	1	1	1	1	1	1	1	61	TRUE*
*Mesorhizobium*	3	7.5	12	13	14	39	40	41	43.5	46	31	35.5	40	41	42	59	TRUE*
*Bacillus*	10	10.5	11	13.5	16	132	157	182	229	276	178	182.5	187	192.5	198	58	TRUE*
*Bogoriella*	1	1	1	1	1	1	1	1	1	1	20	25	30	39	48	58	TRUE*
*Brevibacterium*	25	34.5	44	51.5	59	234	243.5	253	263.5	274	470	478	486	498.5	511	58	TRUE*
*Micrococcus*	1	1	1	1	1	14	15	16	16.5	17	15	19.5	24	27.5	31	51	TRUE*
*Escherichia-Shigella*	81	109.5	138	179	220	634	660	686	922.5	1159	1077	1121	1165	1296	1427	50	TRUE*
*Microbacterium*	3	11.5	20	35.5	51	146	146.5	147	171.5	196	134	166	198	235.5	273	49	TRUE*
*Achromobacter*	17	28.5	40	57.5	75	392	461.5	531	597	663	162	266	370	445.5	521	48	TRUE*
*Corynebacterium*	97	98.5	100	100.5	101	346	381.5	417	432.5	448	943	997.5	1052	1113.5	1175	48	TRUE*
*Acinetobacter*	153	186	219	233	247	487	725.5	964	974	984	1446	1788	2130	2512	2894	47	TRUE*
*Thiovulum*	49	49	49	50	51	1	1	1	1	1	1	1	1	1	1	46	TRUE*

*Indicates statistical significance; the higher the W value the more significant the differences in corresponding bacterial abundance among different groups.

**FIGURE 3:**
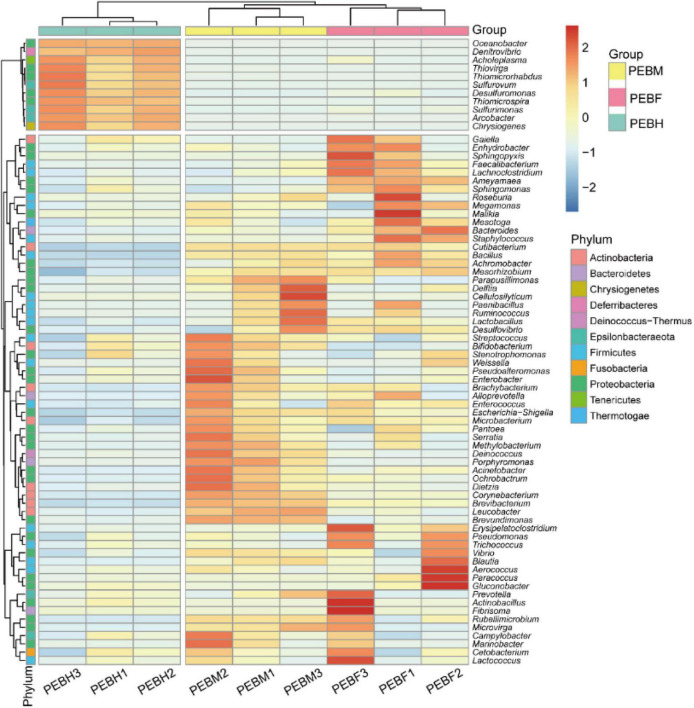
Heatmap and dendrogram of abundant bacterial genera (relative abundance > 0.1%) in the microbial community of samples. Color blocks represent the relative abundance. More red color indicates a higher relative abundance.

Heatmap (Figure 3) showed that the bacteria genera differed in the cultivation bags collected at different growth periods of the *P. eryngii* hyphae. The relative abundances of nine bacteria genera in *P. eryngii* hyphae bags, including *Sulfurimonas*, *Gluconobacter*, *Desulfuromonas*, *Actinobacillus*, *Fibrisoma*, *Gaiella*, *Sphingopyxis*, *Acholeplasma*, and *Ameyamaea*, were decreased sharply or even disappeared. Figure 3 also showed a high degree of similarity between the PEBF and PEBM samples. For example, 11 genera including *Oceanobacter*, *Denitrovibrio*, *Acholeplasma*, *Thiovirga*, *Thiomicrorhabdus*, *Sulfurovum*, *Desulfuromonas*, *Thiomicrospira*, *Sulfurimonas*, *Arcobacter*, and *Chrysiogenes* were obviously more abundant in PEBH than other two periods (Figure 3).

Regarding prokaryotic communities, *Pseudomonas, Acinetobacter*, *Escherichia-Shigella*, *Ochrobactrum*, and *Lactobacillus* were dominant in the microbiome of *P. eryngii* hyphae bags. Still, the bacterial communities varied at different growth periods, and the relative abundance of *Pseudomonas* (Proteobacteria) was raised during the PEBF period. The genus *Pseudomonas* is known as “fungiphills” (Warmink et al., 2009; Zagriadskaia et al., 2013). Warmink et al. (2009) demonstrated that the selection of fungiphills depends on their ability to utilize organic substrates from the fungi exudates. Moreover, the growth of the pathogenic genus *Arcobacter* (Epsilonbacteraeota) could be inhibited by organic acids, such as citric acid and lactic acid (Phillips, 1999). At PEBF, the acid-forming bacteria, such as the genera *Pseudomonas, Acinetobacter*, *Escherichia-Shigella*, *Bacteroides, Ochrobactrum*, *Lactobacillus*, and so on, increased significantly and became the dominant genera. As a result, the genus *Arcobacter* with a significant abundance (40.31%) at PEBH was almost disappeared during the PEBF period. Besides, the identified species of *Pseudomonas*, including *Pseudomonas putida*, which is a growth-promoting inoculant for *A. bisporus* (Zarenejad et al., 2012; Siyoum et al., 2016), may also contribute to promoting the growth of *P. eryngii* hyphae.

After the growth of *P. eryngii* hyphae to fill the cultivation bags, Proteobacteria (41.85%) was still one of the dominant phyla. In comparison, Firmicutes (22.91%), Actinobacteria (12.96%), and Bacteroidetes (8.81%) replaced Epsilonbacteraeota (44.30%) and became the dominant phyla. Bacteria communities in *P. eryngii* hyphae bags are different from those in the soil beneath other edible fungi. Actinobacteria, Chloroflexi, and Proteobacteria were found to comprise the core microbiome from the soils beneath *Morchella sextelata* (Benucci et al., 2019). Three bacteria phyla, Proteobacteria, Acidobacteria, and Actinobacteria, were dominant in the soil samples from the forestland where *Stropharia rugosoannulata* (wine-cap mushroom) (Gong et al., 2018) and other 16 species of mushrooms were cultivated (Pent et al., 2017). Bacterial communities found in the casing layer of *A. bisporus* were Proteobacteria, Firmicutes, Bacteroidetes, and Actinobacteria (Siyoum et al., 2016), similar to phyla found in cultivation bags of *P. eryngii* hyphae. The abundance of Proteobacteria was stimulated by the high nutritional status of soil, while Acidobacteria preferred low nutritional status (Siyoum et al., 2016; Pent et al., 2017; Gong et al., 2018). Although most of the bacterial phyla observed in cultivation bags of *P. eryngii* hyphae and casing layer were very similar, their relative abundances in genera differed significantly between these two habitats. The bacterial population varied even in the casing layers of *A. bisporus* from different mushroom farms (Siyoum et al., 2016; Vieira and Pecchia, 2018; Li et al., 2019). But a general agreement of the distribution of functional categories and different bacterial communities among different composts has also been confirmed (Martins et al., 2013).

### Analysis of Beta Diversity

**FIGURE 4:**
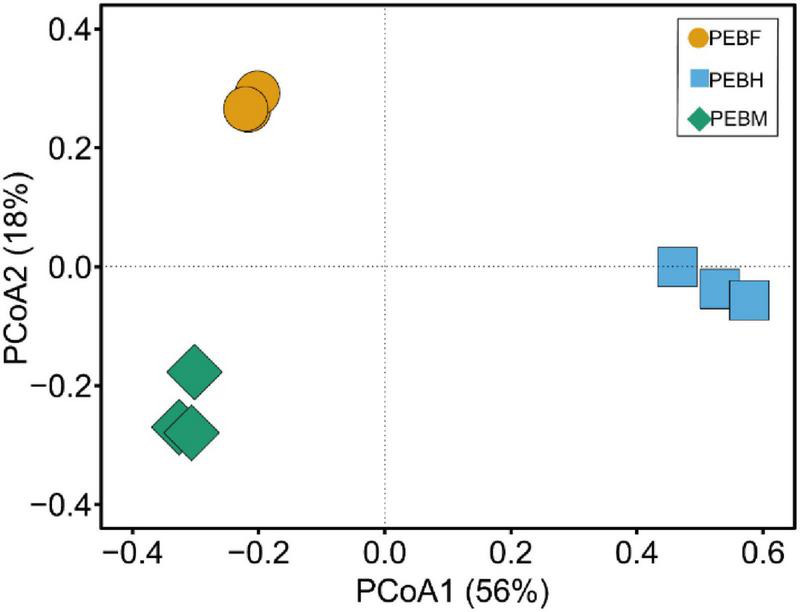
Principal coordinate analysis (PCoA) plot showing the clustering of bacterial communities based on Bray-Curtis distances between samples. The values for the two axes are the percentages of variations attributed to the corresponding axis.

The beta diversity was analyzed by PCoA, and the percent variability for each principal component is PCoA1: 56% and PCoA2: 18%. As shown in Figure 4, ordinations based on Bray-Curtis metric demonstrated a clear separation among the three periods. Thereinto, the distance between the samples PEBH and PEBF was longer than that between the samples of PEBF and PEBM, indicating that the bacterial communities in the PEBF samples were more similar to those at the period of PEBM.

### Effects of *Bacillus cereus* Bac 1 Fermentation Broth on the Growth of *Pleurotus eryngii* Hyphae

The well-grown bacteria were isolated from *P. eryngii* hyphae and identified by 16S rRNA sequencing and physiological and biochemical indexes to explore promoting effects of the eight most dominating bacterial genera on the growth of *P. eryngii* hyphae. The results showed that Bacillus species became the dominant microbiota, and the isolated strain belonged to the *Bacillus cereus* group with a similarity of 100%. The strain was labeled as *Bacillus cereus* Bac 1 and then was subjected to submerged fermentation.

**FIGURE 5:**
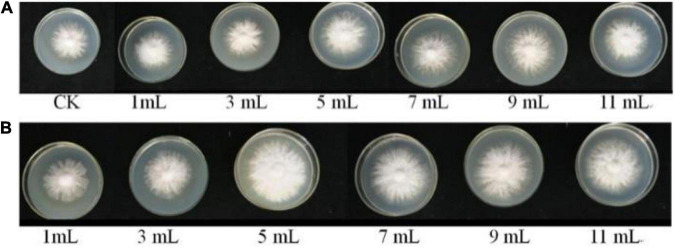
Mycelial morphology of *P. eryngii* cultured in media with the different volumes of LB medium **(A)** and *B. cereus* Bac1 fermentation broth **(B)**, respectively.

**FIGURE 6:**
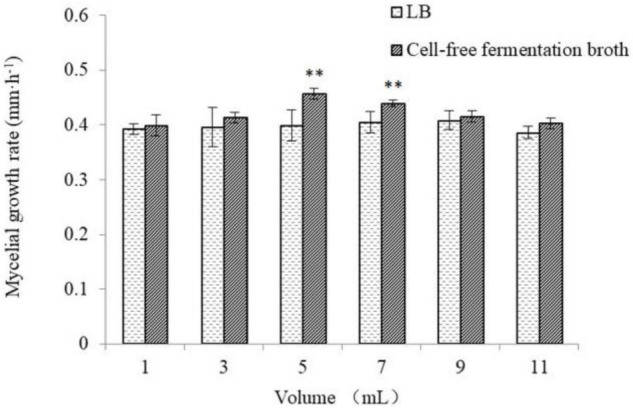
Mycelia growth rate of *P. eryngii* cultured in media with and without the different volumes of *B. cereus* Bac1 fermentation broth. Different letters mean a significant difference (^**^P < 0.01).

As shown in Figures 5, 6, the different volumes of *B. cereus* Bac 1 fermentation broth could promote the growth of *P. eryngii* hyphae. The mycelia growth rate was significantly faster in the modified media containing the 5-mL fermentation broth of *B. cereus* Bac1 than the control ones (*P* < 0.01). The mycelia growth rate reached 0.46 mm/h, which is 1.15-fold higher than the corresponding control. The mycelia cultured in the modified media were stronger and thicker than the control ones. The results showed that the addition of *B. cereus* Bac1 fermentation broth to culture media was beneficial for the growth of *P. eryngii* hyphae. The composition analysis of *B. cereus* Bac1 fermentation broth performed by UHPLC-QTOF-MS technology showed that the extracellular metabolites were mainly classified into antagonistic substances, promoting materials and cyclopeptides, including carboxylic acids and derivatives, organic oxides, benzene, and substituted derivatives, hydrazine and its derivatives, pyrimidine nucleotides, phenols, imidazopyridines, and fatty acyl. These compounds are associated with the growth and development of *P. eryngii*.

Interestingly, the cyclopeptides in the cell-free fermentation broth of *B. cereus* Bac1 promoted the mycelium development of *P. eryngii* as supplemental nitrogen. In addition, the indole acetic acid in the fermentation broth of *B. cereus* could also increase the mycelium growth of *P. eryngii*. However, when a volume of 7–11 ml was used, the inhibiting effects of antagonistic substances became prominent, which decreased the positive effect of the B. cereus cell-free broth on the growth of *P. eryngii*.

### Effects of *Bacillus cereus* Bac 1 Fermentation Broth on the Mycelial Pellet Density of *Pleurotus eryngii*

The effects of *B. cereus* Bac 1 fermentation broth on the mycelial pellet density of *P. eryngii* were analyzed. The results indicated that the mycelial pellet density was increased in the modified media containing less than 9-mL fermentation broth (Table 3). The mycelial pellet density is expressed as the number of mycelial pellets (NMP) per milliliter. The mycelial pellet density reached 44.33 NMP/mL in the modified media containing 5-mL fermentation broth of *B. cereus* Bac1, significantly higher than in the control groups (*P* < 0.01). The higher pellet density indicates that the mycelia grow well with more growing points. The addition of 5-mL *B. cereus* Bac1 fermentation broth to culture media could effectively promote the growth of *P. eryngii* hyphae.

**TABLE 3:** Effects of *B. cereus* Bac 1 fermentation broth on mycelial pellet density.

Samples		Pellet density (NMP/mL)*			Average ± SD	Significant differences (*P* < 0.01)
		I	II	III		
Control 1	CK	28	27	26	27.00 ± 0.58	hij
Control 2	LB-1 mL	25	26	27	26.00 ± 0.58	ij
	LB-3 mL	31	32	33	32.00 ± 0.58	defg
	LB-5 mL	37	36	35	36.00 ± 0.58	bc
	LB-7 mL	32	31	33	32.00 ± 0.58	defg
	LB-9 mL	30	28	28	28.67 ± 0.67	ghi
	LB-11 mL	26	29	26	27.00 ± 1.00	hij
	LB-20 mL	28	25	25	26.00 ± 1.00	ij
In modified media containing different volumes of fermentation broth	FF-1 mL	34	33	32	33.00 ± 0.58	cdef
	FF-3 mL	35	37	36	36.00 ± 0.58	bc
	FF-5 mL	45	45	43	44.33 ± 0.67	a
	FF-7 mL	35	35	38	36.00 ± 1.00	bc
	FF-9 mL	35	33	34	34.00 ± 0.58	cde
	FF-11 mL	23	29	30	27.33 ± 2.19	hi
	FF-20 mL	15	15	16	15.33 ± 0.33	k

*Number of mycelial pellets/mL.

### Effects of *Bacillus cereus* Bac 1 Fermentation Broth on the Laccase and Amylase Activities of *Pleurotus eryngii*

**FIGURE 7:**
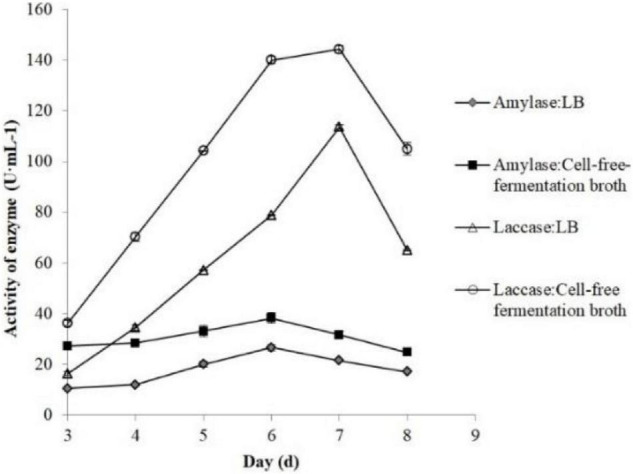
Effects of the different volumes of *B. cereus* Bac1 fermentation broth on laccase activity (△ and _°_) and amylase activity (◆ and ■) of *P. eryngii.*

According to our former research (Sun et al., 2011), we found that the activity of laccase and amylase in edible fungi and their growing cycles were closely related. The edible fungi strains with short growing cycles originated from their high secreted laccase and amylase activity levels. Therefore, the amylase and laccase produced by *P. eryngii* were investigated to demonstrate the effects of *B. cereus* Bac 1 fermentation broth on the enzyme activity. As shown in Figure 7, the enzymatic activities of laccase and amylase were significantly increased by adding Bac 1 fermentation broth. The laccase activity reached a maximum of 144.17 U/mL, 1.27-fold higher than that of the corresponding control. Compared to the control group (113.61 U/mL), The amylase activity was enhanced by 43.83% and reached 38.16 U/mL. These results demonstrated that the Bac1 fermentation broth could significantly increase two extracellular enzymatic activities and further promote the mycelial growth of *P. eryngii*. In general, decreasing the carbon-to-nitrogen ratio in the growth substrate by adding nitrogen sources can promote fungi to produce ligninolytic enzymes, especially laccase (Brezani et al., 2019). The composition analysis of *B. cereus* Bac1 fermentation broth showed that the extracellular metabolites were mainly classified into antagonistic substances, promoting materials, and cyclopeptides. The cyclopeptides in the cell-free fermentation broth of *B. cereus* Bac1 increased nitrogen supply, thus decreased carbon-to-nitrogen ratio in the growth substrate, which stimulated two extracellular enzymatic secretions and activity.

Although fungi have coexisted and interacted with bacteria since the earliest stages of fungal evolution, we still understand relatively little about their interactions (Caporaso et al., 2010). In this study, a *Bacillus cereus* strain was isolated from the cultivation bags of *P. eryngii. B. cereus* strain was reported to improve the heavy metal stress tolerance of its hosts (Azcón, 2010), promote growth (Wu et al., 2012), and inhibit pathogenic bacteria and fungi, such as *Rhizoctonia solani* (Pleban et al., 1997), *Fusarium oxysporum*, *Fusarium solani*, and *Pythium ultimum* (Chang et al., 2007). The results showed that the Bac1 fermentation broth could promote the mycelial growth and extracellular enzyme secretion of *P. eryngii*, demonstrating the mutualistic symbiosis relationship of the fungal host *P. eryngii* and microorganisms during cultivation of *P. eryngii* (Caporaso et al., 2010).

## Conclusion

The study on the composition and dynamics of bacterial communities during *P. eryngii* cultivation and the growth promotion ability of isolated bacteria will provide an important theoretical basis for a deeper understanding of bacteria-fungi interactions, which will also make mushroom science research richer and support the development of medium-term and long-term strategies to increase both profitability and the greening of the industry.

## Data Availability Statement

Raw sequences can be accessed in the Short Read Archive of NCBI under project no. PRJNA801108.

## Author Contributions

LC and MY designed and carried out some experiments. XQ performed the bioinformatics analysis. ZY and YX participated in some experiments. TW and JC collected and prepared samples for sequencing. SS represented the conceptualization and emphasized the project management. All authors read and approved the final manuscript.

## Conflict of Interest

The authors declare that the research was conducted in the absence of any commercial or financial relationships that could be construed as a potential conflict of interest.

## Publisher’s Note

All claims expressed in this article are solely those of the authors and do not necessarily represent those of their affiliated organizations, or those of the publisher, the editors and the reviewers. Any product that may be evaluated in this article, or claim that may be made by its manufacturer, is not guaranteed or endorsed by the publisher.
